# Nongestational ovarian choriocarcinoma with bilateral teratoma: A rare case report and literature review

**DOI:** 10.1097/MD.0000000000036996

**Published:** 2024-05-03

**Authors:** Xue Ao, Sha Hu, Shiqiao Tan, Wei Xiong

**Affiliations:** a Department of Obstetrics and Gynecology, West China Second University Hospital, Sichuan University, Chengdu, China; b Department of Ultrasonic Medical, West China Second University Hospital, Sichuan University, Chengdu, China; c Key Laboratory of Birth Defects and Related Diseases of Women and Children (Sichuan University), Ministry of Education, Chengdu, China; d Reproductive Endocrinology and Regulation Laboratory, West China Second University Hospital, Sichuan University, Chengdu, China; e The Joint Laboratory for Reproductive Medicine of Sichuan University—The Chinese University of Hong Kong, Chengdu, China.

**Keywords:** Case report, choriocarcinoma, laparoscopy, nongestational, ovary, teratoma

## Abstract

**Introduction:**

Trophoblastic neoplasms are often associated with pregnancy, and nongestational trophoblastic neoplasms are extremely rare. Nongestational ovarian choriocarcinoma (NGCO) is a highly aggressive germ cell-derived tumor frequently presenting with early hematogenous metastasis.

**Patient concerns:**

Herein, we report a case of a 28-year-old unmarried woman with regular menstruation who experienced vaginal bleeding 1 week after her last menstrual cycle. Doppler ultrasound revealed bilateral adnexal masses and elevated serum human chorionic gonadotropin (hCG) levels. The patient was initially misdiagnosed as presenting an ectopic pregnancy.

**Diagnosis:**

The final pathology confirmed an International Federation of Gynecology and Obstetrics stage IA NGCO with bilateral mature teratoma of the ovary. This is an extraordinary instance of ovarian choriocarcinoma which emerged without any prior gestation, and the patient’s lack of a history of pregnancy made the diagnosis ignored.

**Interventions:**

After initial surgery and 1 cycle of bleomycin, etoposide, and cisplatin (BEP) chemotherapy, a laparoscopic fertility-preserving comprehensive staging surgery was performed. Two cycles of chemotherapy with BEP were administered as supplemental therapy postsurgery, and leuprorelin was administered to protect ovarian function.

**Outcomes:**

Menstruation resumed 4 months after chemotherapy completion, and tumor indicators were within the normal range. No signs of recurrence were observed at the 36-month follow-up.

**Conclusion:**

NGCO should be considered if a female patient exhibits irregular vaginal bleeding and masses in the adnexal area. The present case and our literature review also highlighted that fertility-sparing surgery and multidrug chemotherapy are effective methods for treating NGCO.

## 1. Introduction

Ovarian choriocarcinomas can be divided into 2 types based on origin. The first type can result from ectopic ovarian pregnancy, ovarian hydatidiform mole, or metastatic ovarian choriocarcinoma, formed by the malignant transformation of gestational trophoblast cells, known as secondary ovarian choriocarcinoma or gestational ovarian choriocarcinoma. The second type frequently originates from ovarian germ cells, unrelated to pregnancy, called primary ovarian choriocarcinoma or nongestational ovarian choriocarcinoma (NGCO), which is extremely rare, with an incidence rate of 1/(3.8 × 10^8^).^[1]^ Typically, reports on NGCO are sporadic or individual. Furthermore, NGCO is a highly aggressive gynecological trophoblastic tumor, prone to early hematogenous metastasis to other organs, and exhibits no significant characteristics during the early stage. Given the abundant human chorionic gonadotropin (hCG) secreted by the tumor, patients often experience menopause or irregular vaginal bleeding. A typical NGCO image presents a mass in the adnexal area with an abundant blood supply. Given the rarity of tumors, this disease is usually ignored or misdiagnosed as ectopic pregnancy, resulting in delayed diagnosis and management. Herein, we present a case of NGCO combined with mature ovarian teratoma, describing the diagnosis, treatment, and prognosis and summarizing experiences of successfully preserving the patient’s fertility. Informed consent was obtained from the patient to publish this case report and accompanying images.

## 2. Case report

**Figure 1.**
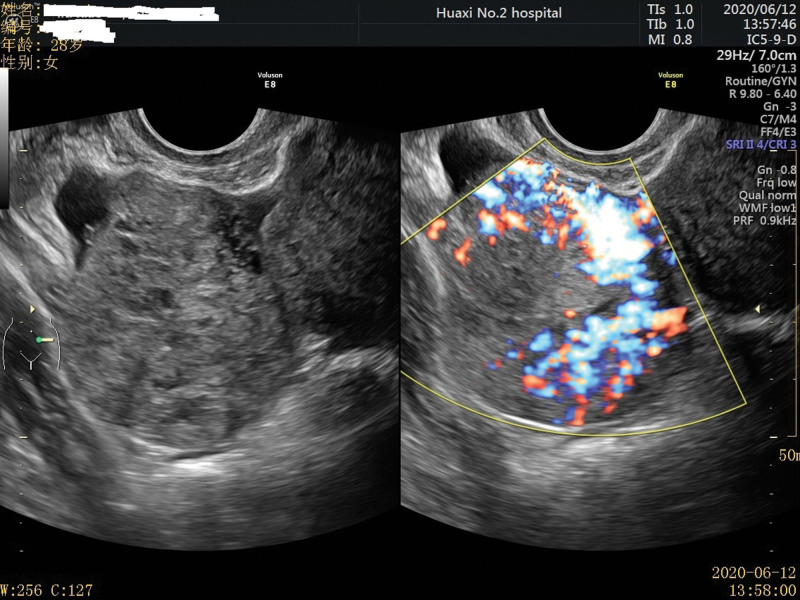
Doppler ultrasound presents the heterogeneous tumor on the left ovary with abundant blood flow signals.

**Figure 2.**
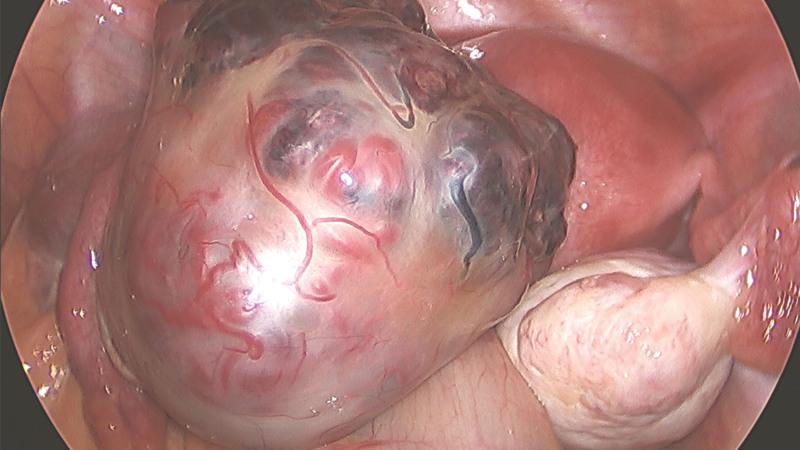
Under laparoscopy, a tumor, 6-cm in diameter, can be observed on the left ovary with uneven, kermesinus surface and dilated blood vessels. The cyst on the right ovary is approximately 4 cm in size and exhibits a smooth appearance.

**Figure 3.**
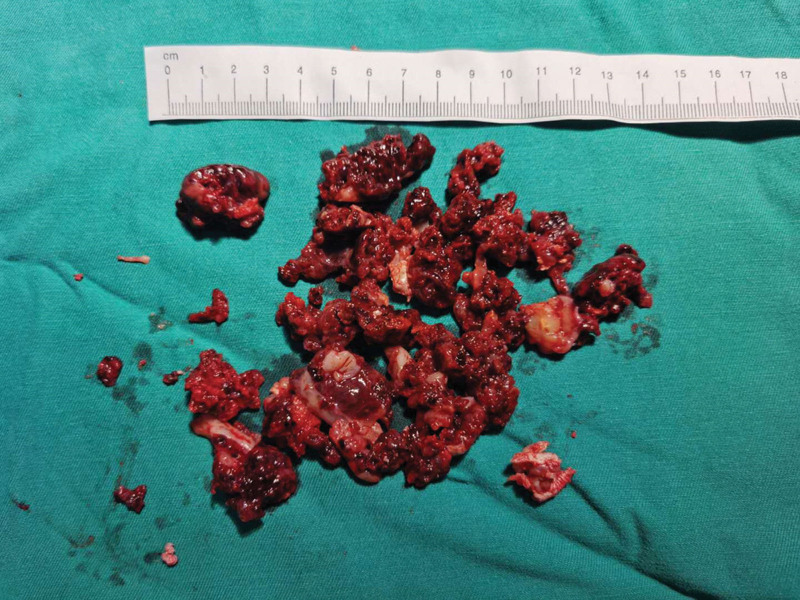
Section of the left ovarian tumor exhibiting kermesinus and spongy surface, with evident hemorrhage and necrosis.

**Figure 4.**
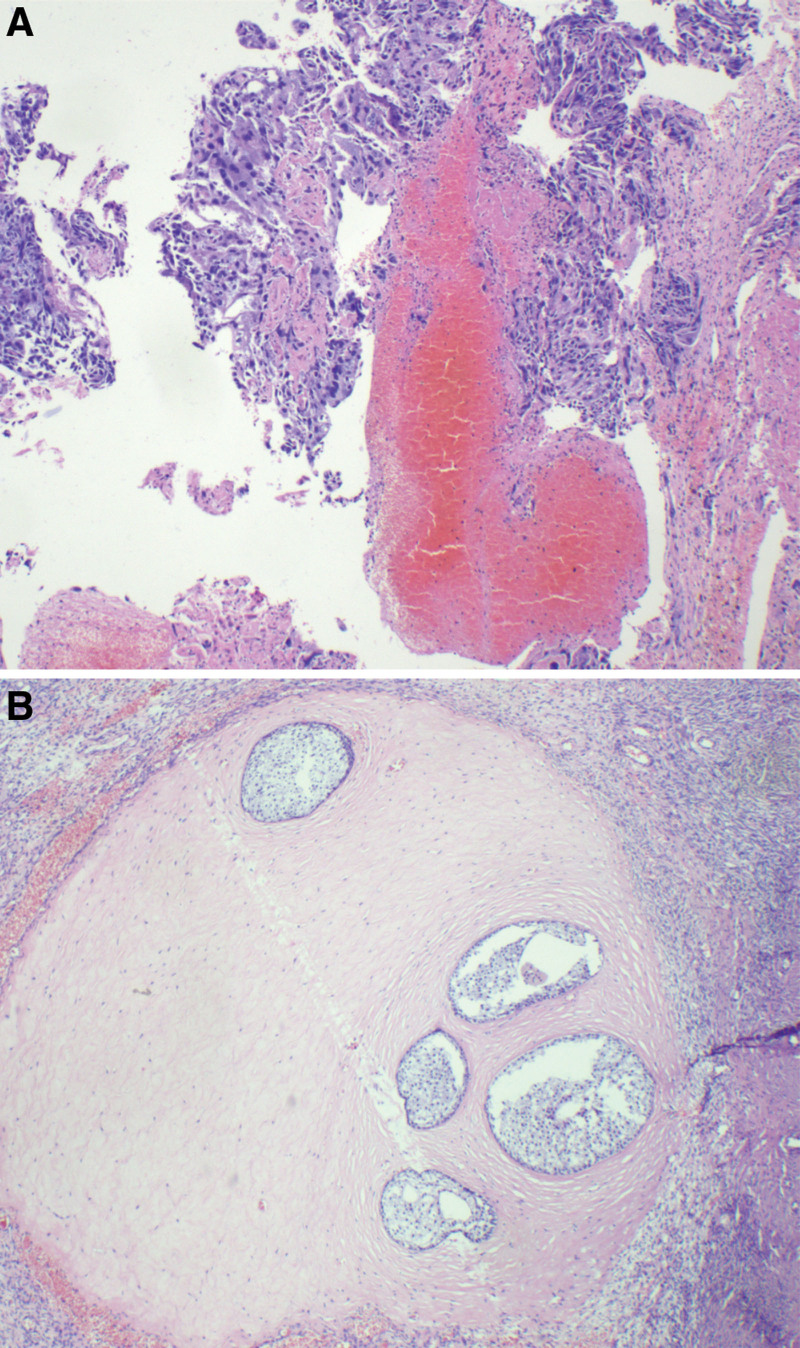
(A) Microscopic appearance of the tumor shows pure choriocarcinoma with widespread necrosis (×200). (B) Mature teratoma component of the left ovary (×200).

**Figure 5.**
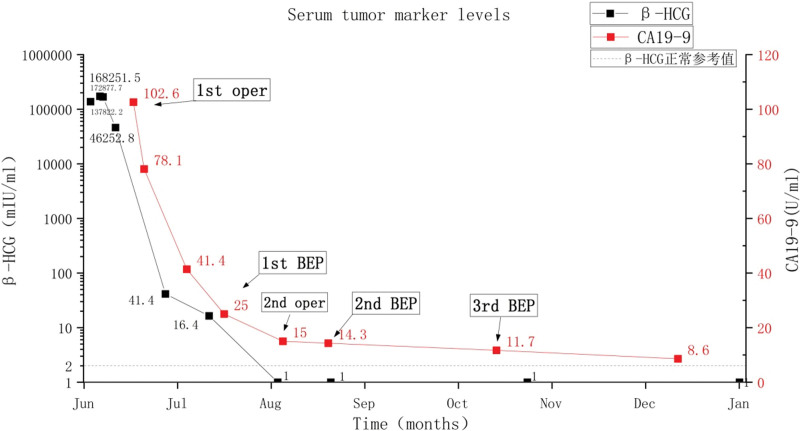
Decrease in serum levels of human chorionic gonadotropin (hCG) and carbohydrate antigen 19-9 (CA 19-9) postsurgery and combination chemotherapy with bleomycin, etoposide, and cisplatin.

A 28-year-old unmarried woman with regular menstruation reported vaginal bleeding 1 week after her last menstrual cycle. The initial serum hCG level was 44,681 mIU/mL during the first examination performed at another hospital. Color Doppler ultrasound revealed a teratoma approximately 3.8 × 3.4 cm^2^ in size in the right adnexa area. A hypoechoic mass, 3.1 × 2.1 cm^2^ in size, was observed in the left adnexal area, along with a strong adjacent echo, 1.5 × 1.3 cm^2^ in size. The lesion in the left adnexal region was speculated as ectopic pregnancy. The patient had no abdominal pain or other discomfort and was recommended to be hospitalized for surgical intervention. She declined the diagnosis of ectopic pregnancy based on strict contraceptive measures and approached the Reproductive Department of our hospital for further diagnosis and treatment. Ultrasound examination detected a teratoma sized 3.7 × 3.5 × 3.8 cm^3^ in the right ovary. A slightly strong echo, approximately 1.5 × 1.6 × 1.5 cm^3^ in size, was observed in the left ovary, with a distinct boundary and no blood flow signal detected. Another heterogeneous echogenic mass was detected in the left adnexal area, approximately 3.9 × 4.2 × 5.2 cm^3^ in size, along with an unclear boundary between the mass and ovary and abundant blood flow signals (Fig. 1). The slightly strong echo on the left ovary was speculated to be an ovarian teratoma. A heterogeneous echogenic mass in the left adnexal area was considered ovarian pregnancy. The patient exhibited elevated levels of serum hCG (137,822.2 mIU/mL) and carbohydrate antigen 19-9 (CA 19-9) (102.6 U/mL; normal reference value < 30.9 U/mL), along with other tumor markers, such as cancer antigen 125 (CA-125), alpha-fetoprotein, carcinoembryonic antigen, and neuron-specific enolase, which were within the normal range. Given the rarity of clinical features, we invited experts in gynecological oncology, gynecological radiochemotherapy, and medical imaging to discuss this case, supplemented with unenhanced and enhanced multidetector computed tomography images of the head, chest, abdomen, and pelvis. No other lesions were detected. To obtain histological evidence, the patient consented to undergo laparoscopic surgery. No ascites were detected during surgery, and no abnormalities were identified in the uterus, omentum, intestine, diaphragm, liver, or peritoneum. A mass, approximately 6 cm in diameter, was observed in the left ovary, presented as a nodular, uneven, and kermesinus protrusion with dilated blood vessels on the surface (Fig. 2). The tumor exhibited a soft and fragile texture, and sections were kermesinus and spongy, with evident bleeding and necrosis (Fig. 3). Furthermore, the right ovary presented a cyst approximately 4 cm in diameter, smooth in appearance and containing hair and fat. The ovarian tumors were resected, placed in specimen bags, and extracted via the trocar port in the abdomen. Rapid freezing pathological examination revealed the following: <left ovarian tumor> a large number of dysplastic trophoblasts with hemorrhage and necrosis and no precise placental villi were observed; <right ovarian cyst> mature cystic teratoma. On the second day postsurgery, the patient presented reduced hCG and CA 19-9 levels (46,252.8 mIU/mL and 78.1 U/mL, respectively). The final pathology findings postsurgery (Fig. 4) were as follows: <left ovarian tumor> mixed germ cell neoplasms: choriocarcinoma mixed with mature teratoma; <right ovarian cyst> mature teratoma. The Department of Gynecological Oncology and Chemoradiotherapy, along with clinical pharmacists, worked to develop a suitable chemotherapy regimen for this patient: 15, 30, and 15 mg bleomycin on days 1, 4, and 15; 100 mg etoposide on days 1 to 5; 30, 20, 20, 20, and 20 mg cisplatin on days 1 to 5. Goserelin acetate was used to protect ovarian function. The patient developed adverse reactions, such as grade IV bone marrow suppression, oral ulcers, and hair loss, after the first chemotherapy cycle. One month later, serum hCG and CA19-9 levels decreased to normal levels (Fig. 5). Subsequently, a laparoscopic fertility-preserving comprehensive staging surgery was performed: left salpingo-oophorectomy, pelvic lymph node dissection, omentectomy, and appendectomy. Histological examination of specimens from the second surgery, including 31 pelvic lymph nodes, showed no malignant tumor involvement. In addition, the pelvic–abdominal cavity washing fluid was negative, with several mature teratoma components detected in the left ovary. The final diagnosis was stage IA mixed germ cell tumor of the left ovary (nongestational choriocarcinoma of the ovary mixed with mature teratoma). The patient completed 2 cycles of bleomycin, etoposide, and cisplatin (BEP) chemotherapy after comprehensive staging surgery and experienced no significant change in body weight during the 6 months of treatment. She was followed up every 3 months for 2 years postsurgery. Menstruation resumed 4 months after completing the last chemotherapy cycle, and tumor indicators were within the normal range. No signs of recurrence were observed after the 36-month follow-up.

## 3. Discussion

NGCO is an extremely rare malignant germ cell tumor, accounting for 1% of ovarian germ cell tumors, common in adolescents and young women and occasionally detected in postmenopausal women.^[2]^ Herein, we systematically searched the literature written in English published in PubMed, Cochrane Library, and Embase from 2000 to date. In total, 35 cases, including the present case report, are summarized in Table 1, excluded an article published in 2001 reported by Goswami et al^[3]^ about 30 cases of pure NGCO. The mean age of the 35 cases was 26.34 ± 14.15 years old, presenting 17, 15, and 2 left, right, and bilateral tumors, respectively. All cases presented tumors originating in the ovaries, exhibiting elevated hCG levels. In addition, increased CA-125 and lactate dehydrogenase levels were detected in some cases, with no abnormality in alpha-fetoprotein levels. In contrast, CA199 and hCG levels were elevated in the present case report, which were reduced during the medical intervention (Fig. 5). In addition to the present case report, 2 additional articles have reported NGCO complicated with mature cystic teratoma.^[3,4]^ On reviewing the literature, we discovered 3 additional articles reporting 5 cases of nongestational choriocarcinoma accompanied by mature teratoma.^[5–8]^ Although an interesting phenomenon, the association between nongestational choriocarcinoma and mature teratoma remains unclear. Typically, the clinical symptoms of NGCO are nonspecific. Adolescent females could have sexual precocity or amenorrhea, and reproductive-age females could experience menopause, abdominal pain, abnormal vaginal bleeding, or early pregnancy reactions. Moreover, some patients remain asymptomatic. An acute abdomen was detected following tumor necrosis or rupture. Ultrasonography revealed a mass in the adnexa without a gestational sac in the uterus, which contributed to the initial misdiagnosis, often misdiagnosed as ectopic pregnancy, ovarian mass pedicle torsion, hemorrhagic ovarian cyst, oviduct ovarian abscess, or even other malignant ovarian tumors.^[9]^ Overall, 10 of the 35 cases were misdiagnosed, of which 7 were misdiagnosed as ectopic pregnancies, including the present case report. In general, hCG in ectopic pregnancies rarely exceeds 10 kU/L, and the probability of hCG levels exceeding 40 kU/L during ectopic pregnancies (excluding live fetuses) was extremely limited.^[10]^ Therefore, physicians should consider the possibility of choriocarcinoma in an ectopic pregnancy with abnormally high hCG levels. More importantly, tumor blood supply, as observed by imaging, should be closely monitored. Generally, the blood flow signal of a common ectopic pregnancy tumor is detected around the tumor. However, choriocarcinomas exhibit abundant blood flow signals within the tumor. The tumor appearance could be observed during laparoscopic surgery, and primary choriocarcinoma is typically soft and brittle, along with nodular protrusions, as well as uneven and kermesinus surfaces. Herein, the prepared section showed kermesinus and spongy surfaces, with evident hemorrhage and necrosis. Intraoperative rapid biopsy could be used to distinguish choriocarcinoma from ectopic pregnancy, however, it was unable to distinguish between gestational and nongestational choriocarcinomas. No microscopic differences were detected, both of which consist of cytotrophoblasts, syncytiotrophoblasts, and intermediate trophoblast cells without villi. Therefore, accurately diagnosing NGCO can be difficult, except in patients who are unable to conceive, are sexually immature, or never had sexual intercourse, especially pure ovarian choriocarcinoma. It is important to distinguish whether choriocarcinoma is of gestational or nongestational origin, as it could impact treatment and prognosis.^[11]^ In 1965, Saito et al^[12]^ proposed criteria for the diagnosis of choriocarcinoma outside the uterus: absence of intrauterine lesions; pathological confirmation of choriocarcinoma; the development of hydatidiform mole pregnancy was excluded; and except coexisting normal intrauterine pregnancy. However, the above standards failed to differentiate NGCO from gestational ovarian choriocarcinoma derived from an ovarian pregnancy or metastatic ovarian choriocarcinoma resulting from a neglected spontaneous abortion. In 1992, Fisher et al^[13]^ first reported that locus-specific minisatellite probes could be employed to identify restriction fragment length polymorphisms in deoxyribonucleic acid (DNA) from tumors, patients, and their partners to distinguish gestational and nongestational trophoblastic tumors. No paternal origin existed if the tumor component was derived only from the patient herself, confirming primary nongestational choriocarcinoma, which remains currently in use. Tsujioka et al^[14]^ have reported that the coincidence of the identical allele was <1% and the genetic origin could be determined using 2 to 3 appropriate variable numbers of tandem repeat loci. A greater number of tandem repeat loci for DNA analysis is helpful for accurate diagnosis, but no consensus exists on the number of tandem repeat loci needed. However, DNA analysis is expensive for clinical use. Some researchers have focused on identifying other strategies to distinguish between these 2 types of trophoblastic tumors. Hayashi et al^[15]^ have observed that the tumor cell β2-microglobulin (BMG) antibody was histochemically positive. The authors highlighted that BMG could be used as an immunohistochemical or serum marker to distinguish gestational and nongestational trophoblastic tumors. Given the mechanism of tissue origin, nongestational choriocarcinoma is often accompanied by other germ-cell tumors, such as immature teratoma, endodermal sinus tumor, and dysgerminoma. Considerable accumulated evidence suggests that histological confirmation of the presence of other germ-cell tumors implies a nongestational etiology.^[16–19]^ In addition, Savage et al^[20]^ have investigated 22 cases of choriocarcinoma in female subjects using DNA analysis and concluded that fallopian tube tumors were usually gestational, whereas tumors at other sites (ovary, pelvis) might be nongestational and “should not be assumed to be metastatic from a regressed or occult intrauterine or intraplacental gestational tumor.” In the present case, we did not perform a genetic analysis of DNA polymorphism for comparison, given the high costs. Typical clinical manifestations, histological diagnosis of mixed ovarian germ-cell tumors, and the patient’s clinical history of no previous pregnancy were sufficient to diagnose NGCO in the present patient. Notably, treatment effectiveness in NGCO remains poor compared with that in gestational choriocarcinoma, and surgery combined with multidrug chemotherapy is considered necessary. However, no consensus has been reached regarding various issues, such as fertility preservation, surgical timing, method, and selection of chemotherapy regimens, and useful literature or national standards are still lacking. Typically, NGCO occurs in children and adolescents. The tumor is sensitive to chemotherapy. Accordingly, fertility-sparing surgery and comprehensive staging are feasible if the patient desires to maintain fertility. Patients presenting drug resistance and extensive metastasis can achieve complete remission with high-dose chemotherapy combined with hematopoietic cell transplantation.^[21]^ Inaba et al^[22]^ have suggested that fertility-preserving surgery is feasible in the case of advanced nongestational choriocarcinoma. Xin et al^[23]^ have reported a case of stage IIB NGCO with the comprehensive staging of fertility preservation using laparoscopy and 3 courses of chemotherapy after surgery, which achieved ideal therapeutic effects. In total, 14 cases of nongestational choriocarcinoma were reviewed in this article; 4 patients (28%) underwent minimally invasive laparoscopic surgery with no indication of the stage, and the rest underwent exploratory laparotomy. Laparoscopic surgery did not appear to impact the prognosis. Laparoscopic surgery for NGCO has rarely been reported, and its safety needs further validation. Among the 35 cases listed in Table 1, 34 explained the surgical scope, among which 21 patients underwent fertility-sparing surgery, and 2 progressed and died due to distant metastasis prior to surgery, in the remaining cases, tumor recurrence had not been observed up to the point of reporting. In addition, chemotherapy regimens, including BEP, EMA/CO, EMA, PVB, and MAC, vary in these cases. Sixteen patients were treated with BEP chemotherapy, one died due to the terminal stage, and the rest achieved ideal efficacy. NGCO often metastasizes early, with hematogenous spread to the lungs (80%), pelvis (20%), liver (10%), and other rare sites, including the gastrointestinal tract, spleen, or kidney.^[24]^ Clinical manifestations of various metastases vary with the site of metastasis. Table 1 shows that 10 cases (28.57%) of NGCO metastasized to the lungs, 7 cases to the pelvic and abdominal cavities, 3 cases to the liver, and the rest to the brain, digestive tract, kidney, adrenal gland, spleen, and bone. To date, the largest retrospective cohort study of nongestational choriocarcinoma included a total of 37 patients, all of whom were Chinese, with a median follow-up period of 41 months; all subjects were treated with surgery and multidrug chemotherapies, with a median of 4.0 courses required to achieve complete remission. The overall complete response rate, relapse rate, and 3-year and 5-year survival rates were 81.1%, 16.7%, 80.0%, and 75.5%, respectively.^[25]^ Gatta et al^[26]^ have reported that BEP completely alleviated ovarian germ-cell tumors and the 5-year relative survival rate was 73%, consistent with the above-reported conclusion. For malignant ovarian germ-cell tumors, BEP systemic chemotherapy was recommended as the preferred regimen according to the NCCN Guidelines Version 2.2020,^[27]^ with a total of 3 cycles required for good-risk patients and 4 cycles for poor-risk patients. In the present case report, both hCG and CA 19-9 levels were reduced to normal levels after 2 months of treatment from the onset to the first chemotherapy postovarian tumor resection and remained normal, with no signs of recurrence.

**Table 1:** Details of 35 cases of nongestational ovarian choriocarcinoma (our case included).

Case	Authors (Ref)	Age (yr)	Presenting complaints	Side	Tumor size (cm)	HCG (mIU/mL)	Other tumor markers	Surgery	R0	Stage^a^	Hyphology	Distant metastases	Diagnostic basis	Chemotherapy	Outcome
1	Our case	28	Vaginal bleeding	Left	6	137,822.2	CA19-9 102.6 U/mL	LSO, ROCE	Yes	IA	Mixed, mature teratoma	None	Histopathology	BEP 3 cycle	NED 20 months
2	Nishino et al^[28]^	28	Headache, nausea, visual field defect, vaginal bleeding	Left	5.5	5030	All negative	TAH, BSO, LSE, BTR	No	IVB	Pure	Brain, lung	DNA analysis	EMA;BEP;EMA/CO;et al	DOD 21 months
3	Lee et al^[29]^	15	Vaginal bleeding	Right	8.5 × 6.3	76,600	NS	RSO, OE, AE	Yes	IIA	Pure	None	Virgin	EMA-CO 3 cycles	NED 60 months
4	Anjum et al^[30]^	36	Pain in abdomen, dyspnea, cough	Left	8.6 × 8.2	6000	NS	LOCE, RSO, OE	No	IVB	Pure	Kidney, liver, lung	No pregnancy history 5 years	Bleomycin, vincristine, and etoposide 6 cycles	Died due to cerebral metastasis
5	Peng et al^[31]^	12	Dyspnea, cough, headache, fever	Right	8 × 5 × 4	120,420	LDH 514 IU/L, CA-125 261.3 U/mL	RSO	No	IV	Pure	Lung, brain	Virgin	EMA-CO 3 cycles	NED 3 months
6	Irene et al^[24]^	9	Abdominal distension	Left	17 × 14 × 8	444,900	CA-125 48.32U/mL	TAH, BSO, OE, AE	No	IIIC	Pure	Bowel, lymph nodes	Prepuberty	EMA-CO	under treatment
7	Mascilini et al^[32]^	27	None	Left	3.4 × 2.3 × 3.8	22,000	NS	LSO	Yes	IA	Pure	None	History, histopathology	BEP 5 cycles	NED 60 months
8	Motamedi et al^[2]^	16	Pain in abdomen, vaginal bleeding	Left	8 × 9	144,600	LDH 1239 IU/L	LSO	No	IIB	Pure	Pelvic cavity	Virgin	BEP 4 cycles	under treatment
9	Syed et al^[33]^	38	Amenorrhea	Bilateral	NS	300,000	NS	TAH, BSO	NS	NS	Pure	NS	No pregnancy history 5 years	Yes, details NS	NED 5 months
10	Ahn et a^[34]^	41	Vaginal bleeding, Lump in abdomen	Right	10.9 × 9.9	1400	All negative	LH, LSO, OE, AE, LND	Yes	IA	Pure	None	DNA analysis	BEP 3 cycles	NED 48 months
11	Wang et al^[35]^	13	Pain in abdomen	Left	16 × 15 × 10	2045 (8 d after surgery)	NS	TAH, LSO	No	IV	Pure	Lung, liver, kidneys, spleen	Before menarche	PVB	DOD 4 months
12	Koyanagi et al^[36]^	29	abdominal distension	Right	20	10,800	All negative	TAH, RSO, PB	NS	IV	Mixed, adenocarcinoma	Lung	DNA analysis	EMA 3 cycles, EMA- EP, et al	NS
13	Rao et al^[37]^	26	Pain in abdomen, osphyalgia	Right	NS	8160	NS	RSO, OE, partial splenectomy and right adrenalectomy	NS	IV	Pure	Spleen, right adrenal gland	Histopathology	Bleomycin, vincristine, and etoposide 6 cycles	Brain metastases 24months later, NED 14 months
14	Xin et al^[23]^	23	Pain in abdomen	Left	8	18,000	All negative	LSO, OE, LND, PB	Yes	IIB	Pure	Pelvic cavity	Virgin	BEP 3 cycles	NED 9 months
15	Hayashi et al^[15]^	10	Pain in abdomen, Lump in abdomen	Right	18 × 15 × 10	6600^b^	All negative	RSO	Yes	IIB	Pure	Rectovaginal pouch	Prepuberty	BEP 3 cycles	NED 62 months
16	Heo et al^[1]^	12	Pain in abdomen, vaginal bleeding	Left	3.83 × 3.43	20,257	LDH 421 mg/dL	LSO, OE, PB	Yes	IA	Mixed, dysgerminoma	None	Virgin	BEP 6 cycles	NED 14 months
17	Exman et al^[38]^	24	headache, tachypnea, constipation	Left	11.5 × 9.3 × 7.4	675,713	LDH 628 U/l, CA-125 124.4 U/mL	TAH, BSO, OE	Yes	IIB	Pure	Pelvic cavity	DNA analysis	BEP 4 cycles	NED, details NS
18	Choi et al^[39]^	33	Pain in abdomen, vaginal bleeding	Left	5 × 4	74,612	All negative	LSO, PB	No	III	Pure	Abdominopelvic cavity	Husband vasectomy	EMA 9 cycles	NED 60 months
19	Ramarajapalli et al^[40]^	25	Pain in abdomen, osphyalgia	Right	14 × 9 × 10	8120	All negative	RSO, OE, partial splenectomy and right adrenalectomy	Yes	IV	Pure	Spleen, right adrenal gland	Histopathology	BEP 3 cycles, EP 4 cycles	NED 5 months
20	Lee and Fong^[41]^	12	Pain in abdomen	Left	NS	5823 (after surgery)	All negative	LSO	Yes	IC1	Pure	Tumor ruptur	46, XY pure gonad-al dysgenesis (Swyer syndrome)	BEP 4 cycles	NS
21	Hu et al^[42]^	23	Vaginal bleeding, Pain in abdomen	Right	NS	26,516	All negative	TAH, BSO, LND, OE, AE	Yes	IIIC	Pure	Omentummajus	DNA analysis	BEP 3 cycles	NED 30 months
22	Lv et al^[43]^	48	Vaginal bleeding, lump in abdomen	Right	18 × 15 × 14	7664.3	CA-125 217.3 U/mL	TAH, BSO, OE, AE, LND, PB	Yes	IIIB	Pure	Vermiform appendix, peritoneum	No pregnancy history 22 years	BEP 6 cycles	NED 12 months
23	Gon et al^[18]^	21	Pain in abdomen, lump in abdomen	Right	14.9 × 12.0 × 8.0	279,000	LDH 1595U/mL, CA-125 67.15 U/mL	RSO	Yes	IA	Pure	None	No pregnancy history, histopathology	NS	NS
24	Ozturk et al^[44]^	21	None	Bilateral	13 × 11,14 × 12	1869	CA-125 86 IU/mL	TAH, BSO, OE, AE, LND	Yes	IB	Pure	None	DNA analysis	EMA/CO 9 cycles	NED 12 months
25	Kong et al^[45]^	10	Pain in abdomen	Left	11 × 8	7957 (after surgery)	CA-125 310U/mL	LSO, OE	NS	Ic	Pure	Tumor rupture	Prepuberty	PVB 5 cycles	NS
26	Park et al^[16]^	55	Pain in abdomen, cough	Right	6.1 × 4.8	64,838	All negative	TAH, BSO, PB	Yes	IV	Pure	Lung	Husband died, no sexual contact 10 years	BEP 3 cycles	NED 20 months
27	Chen et al^[46]^	23	Vaginal bleeding	Left	1.5 × 2.2 × 2.0	18,703 (After MTX mono-therapy)	All negative	LSO, LND, PB	Yes	IA	Mixed, dysgerminoma, immature teratoma	None	No pregnancy history, histopathology	EMA-EP 1 cycle and BEP 3 cycles	NED 36 months
28	Oladipo et al^[47]^	60	Lump in abdomen	Right	16 × 11	11,945	CA-125 82 U/mL, LDH 1550 IU/L	NS	NS	IV	Mixed, epithelial ovarian carcinoma	Lung, brain	DNA analysis	Carboplatin, POMB/ACE	DOD
29	Yamamoto et al^[48]^	19	Vaginal bleeding	Left	10 × 8 × 7.5	206,949.7	CA-125 103.6 U/mL	LSO, OE	Yes	IV	Pure	Lung	DNA analysis	EMA 8 cycles	NED 12 months
30	Koo et al^[49]^	33	Vaginal bleeding	Left	14 × 10 × 7	18,5000	CA-125 74.6 U/mL	TAH, BSO, OE, LND	Yes	IC2	Pure	Tumor rupture	DNA analysis	EMA-CO 4 cycles	NED 18 months
31	Pentheroudakis et al^[50]^	65	Lump in abdomen, ascites	NS	NS	1895	CA-125 4001 KU/L	TAH, BSO, OE, AE, cytoreductive	NS	IV	Mixed, primary peritoneal carcinoma, ovarian type	Liver	Histopathology, postmenopausa women	EMA-CO, EMA-EP	DOD 12 months
32	Balat et al^[51]^	24	Vaginal bleeding	Right	5 × 6	45.701	CA-125 85 IU/mL	TAH, BSO, OE, LND	No	IV	Pure	Lung, sternum, centrum	Histopathology	BEP 1 cycle	DOD
33	Goswami et al^[3]^	18	Pain in abdomen, nausea, dyspnea, vaginal bleeding	Left	10 × 12	88,385	NS	LSO, OE, PB, ROCE	Yes	IA	Pure	None	Virgin	MAC 4 cycles	NED 5 months
34	Jain et al^[4]^	33	Pain in abdomen, amenorrhea, virilism	Right	7.8 × 5.6 × 3.5	146	All negative	RSO	Yes	IC3	Mixed, low grade stromal cell tumor, mature teratoma	Peritoneal washings were positive	Histopathology	BEP 3 cycles	NED 18 months
35	Inaba et al^[22]^	12	Vaginal bleeding	Right	11	25,000^b^	All negative	RSO, OE	No	III	Pure	Lung, omentum majus	Virgin	BEP 4 cycles, carboplatin, etoposide, and ifosphamide	NED 12 months

AE = appendectomy, B = bilateral, BEP = bleomycin, etoposide, and cisplatin, BTR = brain tumor resection, DOD = dead of disease, EMA = etoposide, methotrexate and actinomycin-D, EMA/CO = etoposide, methotrexate/cyclophosphamide and vincristine, L = left, LDH = lactate dehydrogenase, LH = laparoscopic hysterectomy, LND = lymph node dissection, LOCE = left ovarian cystectomy, LSE = lung segmentectomy, MAC = methotrexate, actinomycin-D, chlorambucil, NED = no evidence of disease, NS = not stated, O = oophorectomy, OE = omentectomy, PVB = cisplatin, bleomycin, vincristine, R = right, ROCE = right ovarian cystectomy, S = salpingectomy, TAH = total abdominal hysterectomy.

a According to FIGO Staging System,^[27]^ Partly cases on the basis of pelvic lymphadenectomy not done; postoperative CT scan showed no retroperitoneal lymphadenopathy or metastasis.

b Dose unit is ng/mL.

In conclusion, NGCO should be considered in women with irregular vaginal bleeding and masses in the adnexal area. In the present case report, the staging of fertility-preserving laparoscopic surgery when the lesion was confined to the adnexa was reasonable and achieved satisfactory results. A review of 35 cases indicated that the combined treatment with surgical intervention and postoperative chemotherapy afforded an ideal effect.

## Author contributions

**Data curation:** Xue Ao, Wei Xiong.

**Formal analysis:** Xue Ao.

**Resources:** Xue Ao.

**Writing—original draft:** Xue Ao.

**Writing—review & editing:** Xue Ao, Shiqiao Tan, Wei Xiong.

**Methodology:** Sha Hu.

**Validation:** Sha Hu.

**Conceptualization:** Wei Xiong.
